# Proteome-wide Mendelian randomization identifies therapeutic targets for ankylosing spondylitis

**DOI:** 10.3389/fimmu.2024.1366736

**Published:** 2024-03-19

**Authors:** Wenlong Zhao, Peng Fang, Chengteng Lai, Xiaoyu Xu, Yang Wang, Hao Liu, Hui Jiang, Xiaozhou Liu, Jun Liu

**Affiliations:** ^1^ Department of Orthopedics, The Second Affiliated Hospital of Nanjing Medical University, Nanjing, China; ^2^ Department of Orthopedics, The Affiliated Jinling Hospital of Nanjing Medical University, Nanjing, China; ^3^ Department of Orthopedics, Nanjing Jinling Hospital, Affiliated Hospital of Medical School, Nanjing University, Nanjing, China; ^4^ Department of Biology, Wake Forest University, North Carolina, NC, United States

**Keywords:** plasma proteins, ankylosing spondylitis, Mendelian randomization, phenome-wide association study, drug target

## Abstract

**Background:**

Ankylosing Spondylitis (AS) is a chronic inflammatory disorder which can lead to considerable pain and disability. Mendelian randomization (MR) has been extensively applied for repurposing licensed drugs and uncovering new therapeutic targets. Our objective is to pinpoint innovative therapeutic protein targets for AS and assess the potential adverse effects of druggable proteins.

**Methods:**

We conducted a comprehensive proteome-wide MR study to assess the causal relationships between plasma proteins and the risk of AS. The plasma proteins were sourced from the UK Biobank Pharma Proteomics Project (UKB-PPP) database, encompassing GWAS data for 2,940 plasma proteins. Additionally, GWAS data for AS were extracted from the R9 version of the Finnish database, including 2,860 patients and 270,964 controls. The colocalization analysis was executed to identify shared causal variants between plasma proteins and AS. Finally, we examined the potential adverse effects of druggable proteins for AS therapy by conducting a phenome-wide association study (PheWAS) utilizing the extensive Finnish database in version R9, encompassing 2,272 phenotypes categorized into 46 groups.

**Results:**

The findings revealed a positive genetic association between the predicted plasma levels of six proteins and an elevated risk of AS, while two proteins exhibited an inverse association with AS risk (*P*_fdr_ < 0.05). Among these eight plasma proteins, colocalization analysis identified AIF1, TNF, FKBPL, AGER, ALDH5A1, and ACOT13 as shared variation with AS(PPH3+PPH4>0.8), suggesting that they represent potential direct targets for AS intervention. Further phenotype-wide association studies have shown some potential side effects of these six targets (*P*_fdr_ < 0.05).

**Conclusion:**

Our investigation examined the causal connections between six plasma proteins and AS, providing a comprehensive understanding of potential therapeutic targets.

## 1 Introduction

Ankylosing Spondylitis (AS) is a chronic inflammatory autoimmune disease that primarily affects the spine, sacroiliac joints and their adjacent soft tissues (1). In the progression of the disease, it can lead to fibrosis and calcification, ultimately leading to ankylosis of the spine and joints (2). AS has a prevalence ranging from 0.2% to 0.5% and is most commonly observed in young males, with a male-to-female ratio of 3:1 (3). Current treatments, primarily non-steroidal anti-inflammatory drugs (NSAIDs) and biologics like TNF inhibitors, mainly focus on symptom management and are not universally effective for all patients (4, 5). Furthermore, these treatments can have serious side effects and do not halt disease progression or address the underlying causes of AS (6). Therefore, it is crucial to seek more effective strategies that not only alleviate symptoms but also target the underlying pathophysiology of AS, aiming to prevent progression, reduce complications, and improve the quality of life for affected individuals.

Recently, Mendelian randomization (MR) analysis has become a prevalent tool for repurposing licensed drugs and uncovering new therapeutic targets (7, 8). Genome-wide association studies (GWAS) have identified specific single nucleotide polymorphisms (SNPs) on chromosomes that regulate the expression of proteins. These SNPs are proportional to the quantitative traits of protein abundance and are also referred to as protein quantitative trait loci (pQTL) (9). MR uses these PQTL to act as instrumental variables to explore potential causal associations between exposure and outcome to screen for drug targets and biomarkers (10, 11). Compared to observational studies, MR helps alleviate the impact of confounding factors, thereby enhancing the reliable assessment of causal relationships (12). Additionally, with the application of the phenome-wide association study (PheWAS), it is possible to predict the adverse reactions associated with these targets (13).

Plasma proteins play crucial roles in diverse biological processes such as signaling, transportation, growth, repair, and immune defense. Their dysregulation is common in various diseases, making them significant targets for drug development (14). Hence, we conducted an extensive proteome-wide MR investigation to pinpoint potential therapeutic targets for AS. Initially, a two-sample MR analysis was employed to gauge the causal impacts of plasma proteins on AS. Subsequently, we conducted colocalization analyses to validate the reliability of the findings. Lastly, we evaluated the potential adverse effects associated with the identified druggable proteins for AS treatment through PheWAS.

## 2 Methods

### 2.1 Ethical approval and study design

**Figure 1:**
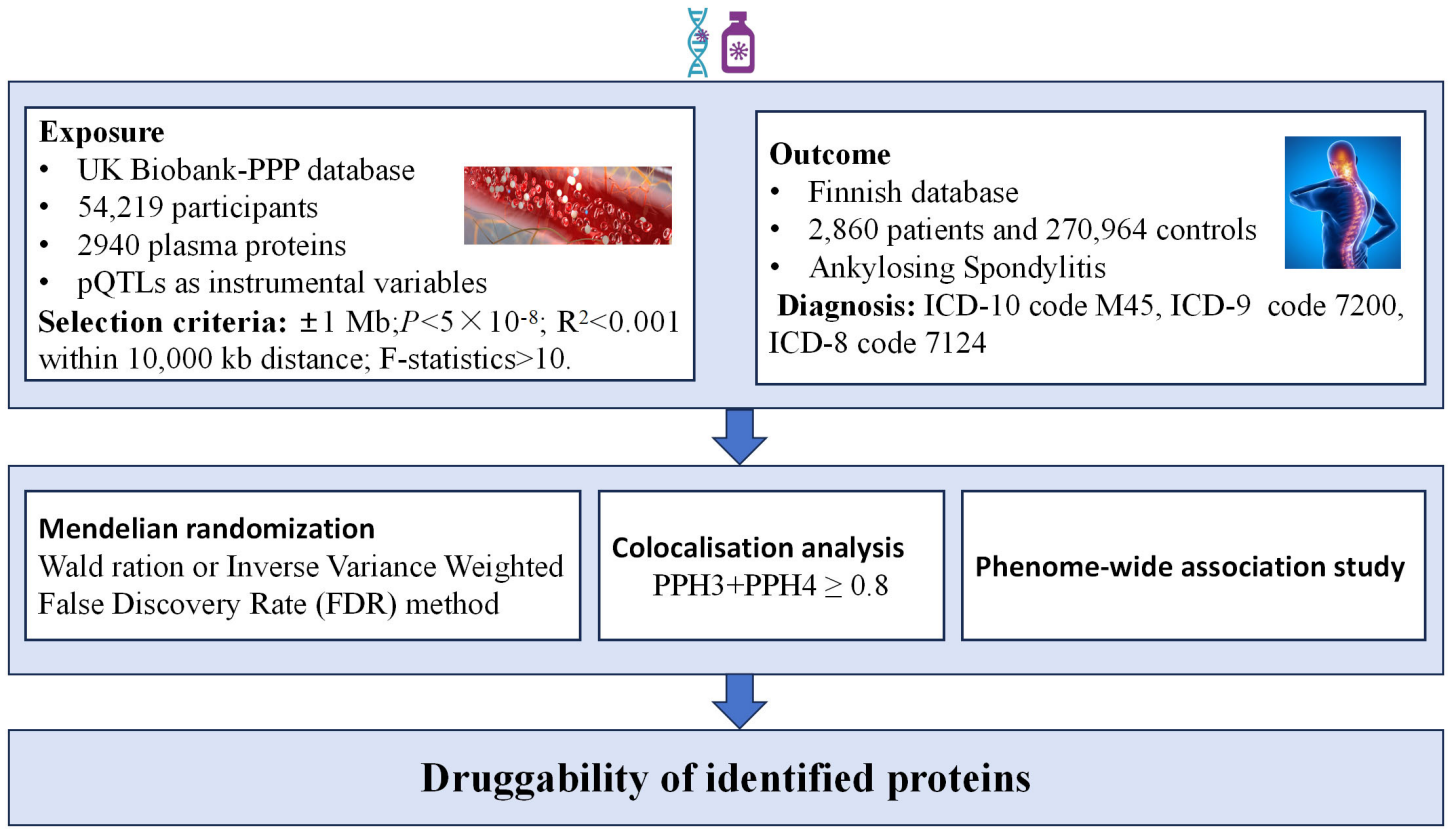
Flowchart of the study design.

In this research, we utilized extensive GWAS summary data from original studies where all participants provided informed consent. Given our reliance solely on aggregate statistical data, no further ethical clearance was needed. The study’s comprehensive design is depicted in **Figure 1**.

### 2.2 Plasma pQTLs

Plasma pQTLs, extracted from the UK Biobank-PPP database, originated from the plasma proteomic profiles of 54,219 UK Biobank participants (https://www.synapse.org/#!Synapse:syn51365303). A thorough mapping of pQTLs was conducted for 2,940 proteins (15). pQTL’s genomic position in relation to a specific protein is often in close proximity to the corresponding gene. Practically speaking, a pQTL near its cognate gene is referred to as a “cis-pQTL,” under the assumption that the pQTL exerts its influence through the cognate gene. Conversely, if a pQTL is situated far from the cognate gene (or on a different chromosome), it is termed a “trans-pQTL,” with the assumption that it operates through an intermediate gene (9). Various studies have employed distinct distance cutoffs to distinguish cis-pQTLs from intrachromosomal trans-pQTLs, with common thresholds being 500 kb or 1,000 kb (16, 17). In the proteome-wide MR study focused on drug targets, we opted for pQTLs as instrumental variables, applying specific selection criteria. These criteria are outlined as follows:1.The SNP within a vicinity of ±1 Mb around the gene region (cis-acting pQTLs); 2.A genome-wide significant threshold of *P*<5×10^-8^ to identify highly correlated SNPs with plasma proteins; 3. To guarantee the inclusion of independent SNPs and mitigate the influence of linkage disequilibrium (LD) on the outcomes, we set a threshold of 0.001 for the linkage disequilibrium parameter (r^2^) and a genetic distance of 10,000 kb; 4. F-value greater than 10 to exclude weak instrumental variable bias (18, 19).

### 2.3 GWAS statistics of AS

AS genetic association research data is sourced from the R9 version of the Finnish database(https://r9.finngen.fi/). The diagnosis of this condition is determined based on the ICD-10 (International Classification of Diseases, 10th edition) code M45, as well as the codes 7200 from ICD-9 (9th edition) and 7124 from ICD-8 (8th edition). The dataset comprises information from 2,860 patients and 270,964 controls.

### 2.4 MR analysis

In this study, we conducted a two-sample MR analysis with plasma proteins as the exposure and AS as the outcome. The selection criteria for pQTLs remained consistent with the standards outlined earlier. For the MR analysis, we utilized the R package “TwoSampleMR” V.0.5.6. When only one SNP was available for a particular protein, we applied the Wald ratio method. Conversely, if there were two or more SNPs available, we employed the inverse variance-weighted (IVW) method (20). Considering our repetitive calculations, we employed the False Discovery Rate (FDR) method for P-value correction. A *P*_fdr_ value below 0.05 was considered statistically significant.

### 2.5 Colocalization analysis

Colocalization analysis aims to confirm the presence of shared causal genetic variants between the exposure and outcome, providing further validation of the MR results. For proteins with positive MR results, we conducted colocalization analysis, examining SNPs within ±1MB range upstream and downstream of the genes corresponding to these proteins (cis-pQTLs), and their colocalization with AS (21). The colocalization analysis involves five hypotheses: H0 indicates that the selected SNP within the locus is unrelated to both protein A and disease B; H1 suggests that the SNP within the selected locus is associated with protein A but not with disease B; H2 implies that the SNP within the selected locus is related to disease B but not to protein A; H3 states that the SNP within the selected locus is associated with either protein A or disease B, but the two are independent SNPs; H4 signifies that the SNP within the selected locus is concurrently associated with both protein A and disease B, and is a shared SNP. Due to limited power in the co-localization analysis, we focused our examination on genes with a combined posterior probability of association (PPH3+PPH4) equal to or greater than 0.8 (22).

### 2.6 Phenome-wide association study

PheWAS, also known as reverse GWAS, is a method utilized to explore associations between SNPs or phenotypes and a wide array of phenotypes spanning the entire phenome (23, 24). This approach is particularly valuable for investigating potential side effects related to drug targets (22, 25). In this study, the exposure was derived from plasma proteins exhibiting positive MR results, and the screening criteria for instrumental variables remained consistent with those described previously. The outcome involved obtaining phenotypic data from the Finnish database in version R9, encompassing 2272 phenotypes categorized into 46 groups. This extensive dataset was employed for phenome-wide MR analysis. A *P*_fdr_ value below 0.05 was considered statistically significant.

## 3 Results

### 3.1 Plasma proteins related to AS

**Figure 2:**
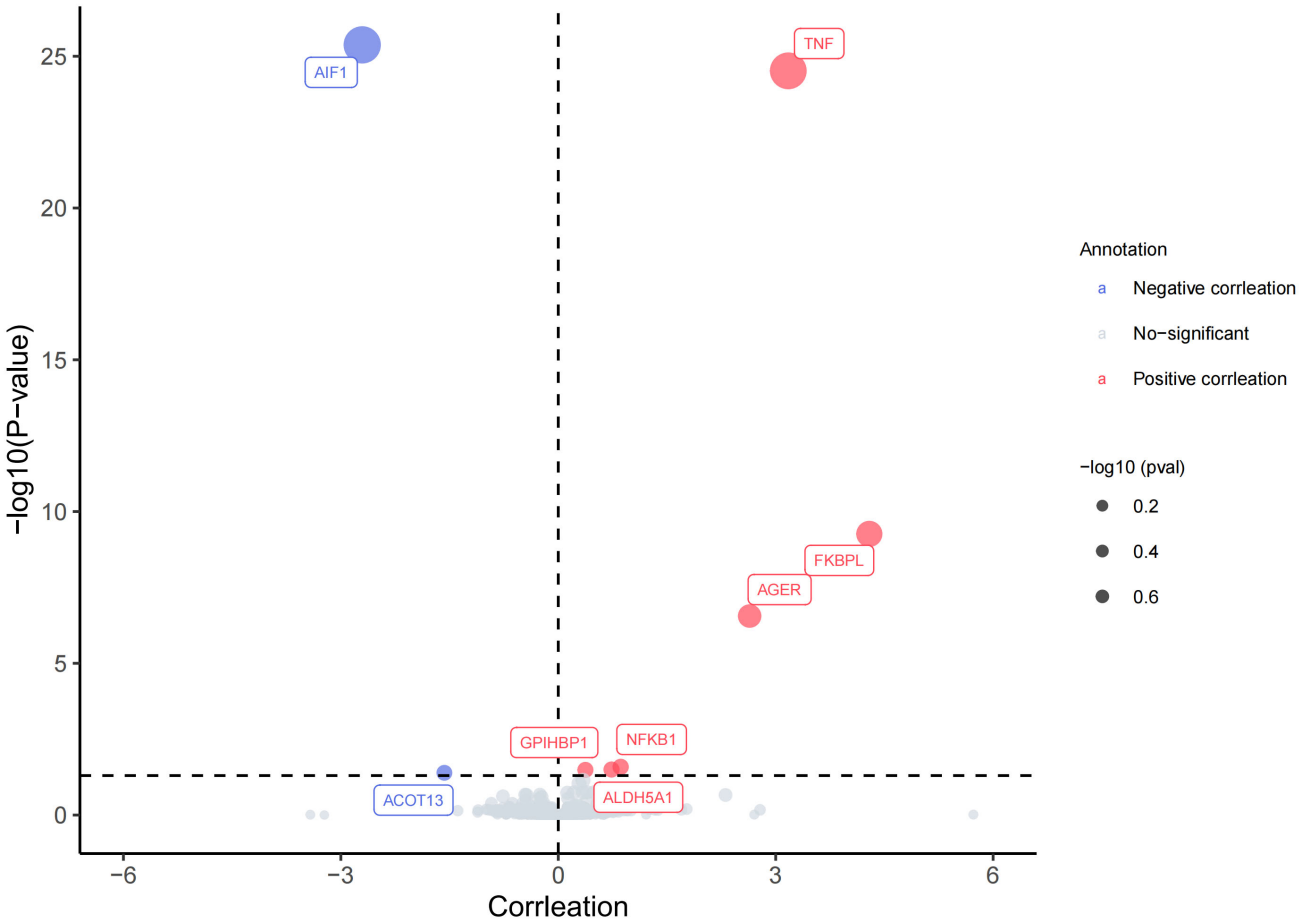
Volcano plot of MR results: Causal relationship between plasma proteins and AS.

**Figure 3:**
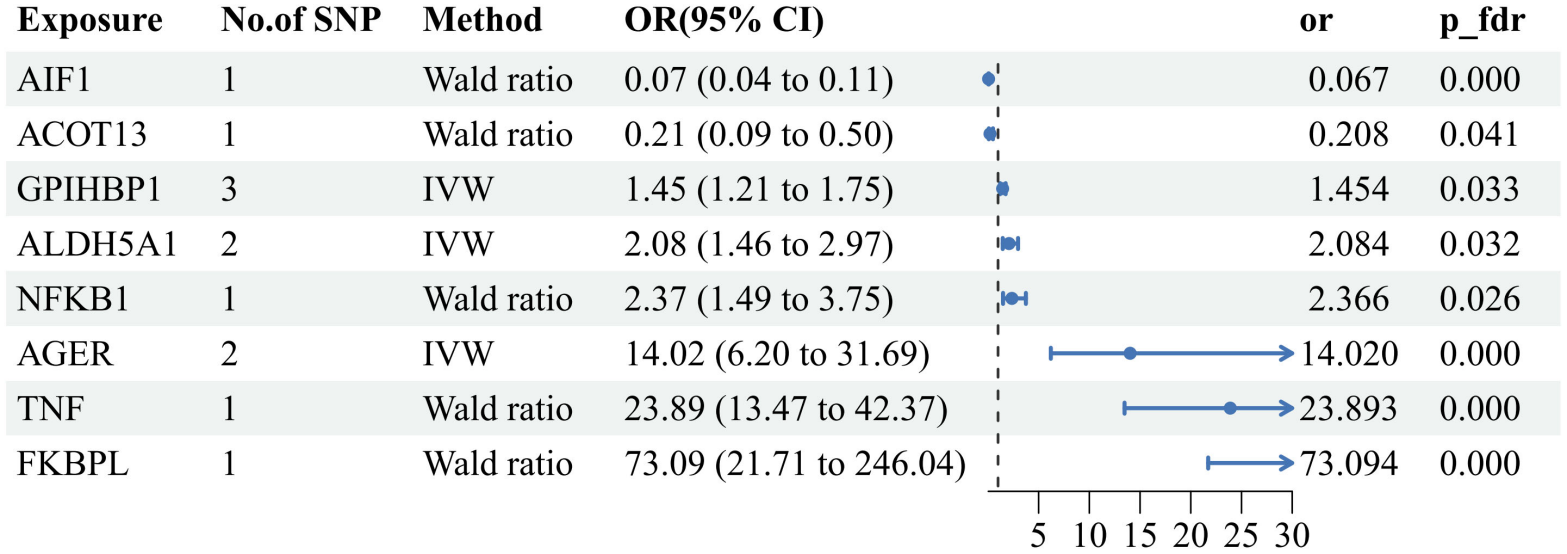
Forest plot of the MR results: Effects of 8 plasma proteins on AS. CI:confidence interval; OR: odds ratio.

Upon strict adherence to the instrumental variables screening criteria in this study, eventually a total of 1908 plasma proteins were included in the MR analysis. Relevant SNP information can be found in **Supplementary 1**. It is worth noting that, among the subset of 1908 plasma proteins, the MR analysis, based on IVW or Wald ratio results (*P*_fdr_<0.05), revealed positive causal associations with AS for six plasma proteins—TNF(tumor necrosis factor), FKBPL (FK506-binding protein-like),AGER(advanced glycation end product-specific receptor),NFKB1(nuclear factor NF-kappa-B p105 subunit), ALDH5A1(succinic semialdehyde dehydrogenase, mitochondrial),and GPIHBP1 (Glycosylphosphatidy inositol-anchored high-density lipoprotein-binding protein 1). The odds ratios and 95% confidence intervals for these associations are 23.893 (95%CI:13.472-42.374), 73.094 (95%CI:73.094-246.038), 14.020 (95%CI:6.203-31.688), 2.366 (95%CI: 1.494-3.745),2.084(95%CI:1.461-2.972) and 1.454(95%CI:1.208-1.750) respectively. In contrast, two plasma proteins—AIF1(allograft inflammatory factor 1) and ACOT13 (acyl-CoA thioesterase 13)—exhibited negative causal associations with AS. The odds ratios and 95% confidence intervals for these associations are 0.067(95% CI:0.041-0.107) and 0.207(95% CI:0.087-0.499). For comprehensive details, please refer to **Figures 2**, **3** and **Supplementary 2**.

### 3.2 Sensitivity analysis for plasma proteins associated with AS

**Figure 4:**
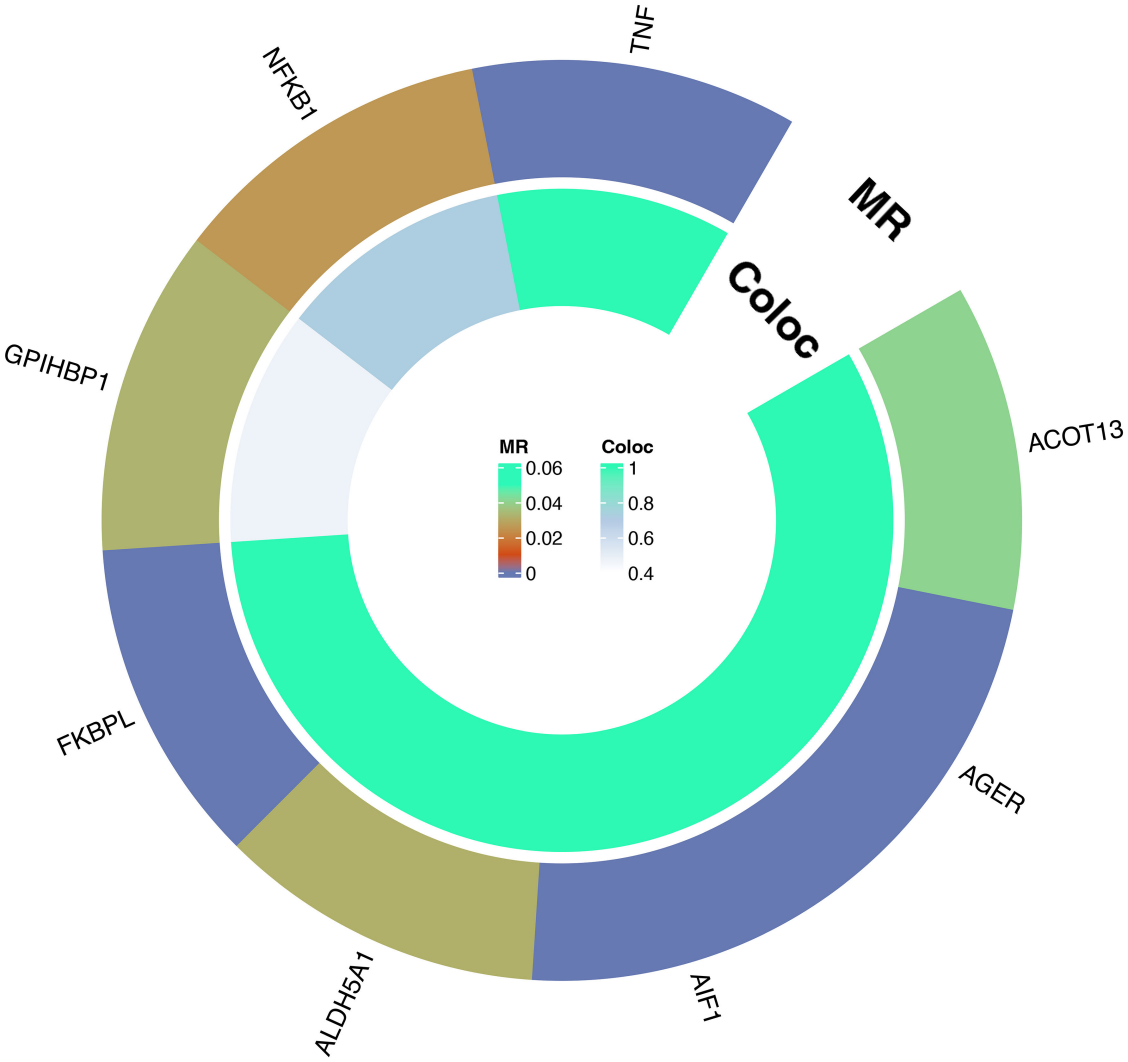
Circle plot of results of MR and colocalization analysis: Causal relationship between plasma proteins and AS.

For these 8 plasma proteins, we conducted gene colocalization analysis within a range of ±1MB upstream and downstream of their respective genes to explore potential associations with AS. The results indicate that AIF1, TNF, FKBPL, AGER, ALDH5A and ACOT13 may share a causal variant in this region (PPH3+PPH4>0.8), while GPIHBP1 and NFKB1 may not share a causal variant with AS in this region (PPH3+PPH4<0.8). For detailed information, please refer to **Figure 4** and **Supplementary 3**. This suggests that these 6 plasma proteins may serve as potential targets for treating AS.

### 3.3 phenome-wide associations analysis for 6 plasma proteins linked to AS.

**Figure 5:**
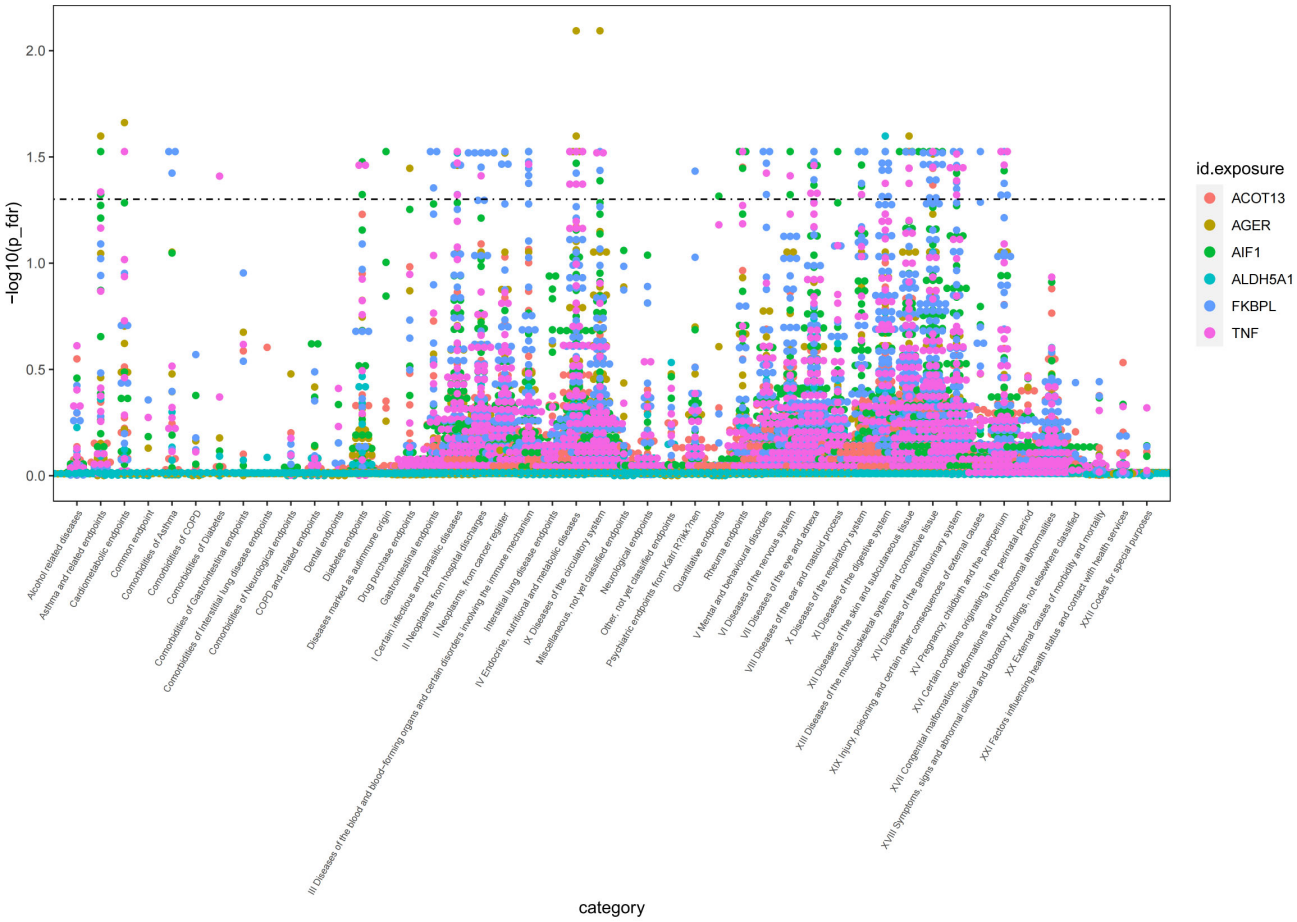
Manhattan plot of result of PheWAS analysis of associations between 6 AS-associated plasma proteins and other disease outcomes.

To evaluate the potential beneficial or deleterious effects of the 6 plasma proteins associated with AS on other phenotypes, we conducted a phenome-wide association analysis, screening 2279 phenotypes across 46 categories from the Finnish database (version R9).We observed significant causal associations between AIF1 and 36 phenotypes across 18 categories (*P*_fdr_<0.05), TNF and 40 phenotypes across 16 categories (*P*_fdr_<0.05), FKBPL and 73 phenotypes across 18 categories (*P*_fdr_<0.05), AGER and 16 phenotypes across 11 categories (*P*_fdr_<0.05), ALDH5A1 and 3 phenotypes across 3 categories (*P*_fdr_<0.05), and ACOT13 and 6 phenotypes across 4 categories (*P*_fdr_<0.05).For detailed data, please see **Figure 5** and **Supplementary 4**. These phenotypes may be therapeutic objects or deleterious effects for the target protein.

## 4 Discussion

AS is a chronic inflammatory disease primarily affecting the spine and sacroiliac joints, potentially leading to spinal stiffness and deformity. In recent years, the emergence of new treatment approaches for AS, especially therapeutic monoclonal antibodies targeting TNF-α and IL-17A, has revealed crucial pathological pathways (26). However, fewer than half of the patients experience substantial improvement in their condition after using TNF-α or IL-17A inhibitors (27). It is worth noting that, since AS currently lacks a cure, the majority of patients require ongoing medication, which may have side effects, to control symptoms (6). Therefore, the identification of new therapeutic targets is paramount for improving patient outcomes.

Plasma proteins with positive correlation with AS in this study were TNF, FKBPL, AGER, and ALDH5A1.TNF is a crucial cytokine playing a key role in the human immune response, involving various inflammatory and autoimmune diseases in their onset and development. Its involvement in AS has been extensively studied. Drugs inhibiting TNF, such as adalimumab, golimumab, infliximab, certolizumab pegol, and etanercept, have shown significant effectiveness in the treatment of AS (5, 28). This further establishes the reliability of our study that TNF as a drug target for the treatment of AS. However, TNF inhibitors still present some challenges, including variability in responsiveness, cost issues, and adverse reactions (6). The PheWAS results of this study appear to support these relatively more serious adverse effects, including basal cell carcinoma, cutaneous malignancies, and chronic nephritis syndrome. Therefore, attention should be paid to these potential adverse reactions when using tumor necrosis factor inhibitors and regular reviews should be performed to ensure early detection and management (6, 29–32). Although observational studies do not appear to support an increased risk of malignancy with tumor necrosis factor inhibitors (33). FKBPL is a newly discovered protein known to be closely associated with anti-angiogenic processes (34). Although its direct association with AS has not been extensively studied, a possible negative correlation can be hypothesized on the basis of available indirect evidence. This hypothesis is based on the central role of angiogenesis in the pathogenesis of AS, particularly its promotion of pathological ossification (35, 36). However, our recent preliminary results from a MR show that there appears to be an unexpected positive correlation between variants in FKBPL and the risk of AS. This finding suggests that the relationship may be more complex than expected, and further in-depth studies are needed to explain this paradox and to fully understand the role of FKBPL in the pathogenesis of AS. Tacrolimus is an inhibitor of FKBPL and functions as an immunosuppressor by binding to the pro-immunoglobin FKBP-12 (FK506 binding protein), creating a new complex that reduces peptidyl-prolyl isomerase activity (37, 38). Besides, based on the results of PheWAS, the potential benefits of FKBPL inhibitors for the treatment of ankylosing spondylitis appear to be much lower than the side effects they may cause (FKBPL inhibitors may cause up to 58 adverse reactions, including tumors, endocrine system, gastrointestinal system, and other related disorders, with a therapeutic effect in only 5 diseases).

AGER, also known as RAGE, is a member of the immunoglobulin superfamily and is characterized by its type 1 transmembrane pattern recognition receptors. Its primary role is to serve as a receptor for advanced glycosylation end products (AGEs) (39). Upon binding of AGEs to AGER, an enhanced inflammatory response is triggered, leading to the upregulation of various pro-inflammatory cytokines, including NF-κB, TNF-α, IFN-γ, IL-22, IL-6, IL-4, and IL-1β (40–42). Moreover, AGEs have the capability to induce macrophage polarization towards the M1 type through activation of the RAGE/ROS/TLR4/STAT1 signaling pathway (40). The M1-type macrophages, known for their pronounced pro-inflammatory properties, significantly contribute to the initiation and progression of AS. Therefore, AGER is poised to become a novel therapeutic target for AS. sRAGE is a soluble form of AGER, which is a variant of AGER produced by shear or protease degradation, and is considered a natural AGER antagonist that modulates AGER-mediated biological effects (41, 43). In addition, according to the findings of the PheWAS study, the utilization of AGER inhibitors may elevate the risk of nine diseases, including heart failure, non-small cell lung cancer, and adrenocortical insufficiency, among others. ALDH5A1 also known as SSADH (succinic semialdehyde dehydrogenase) is an enzyme involved in the metabolic pathway of gamma-aminobutyric acid (GABA) (44). In GABA metabolism, GABA is first converted to succinic semialdehyde by GABA transaminase, and then ALDH5A1 further oxidizes succinic semialdehyde to succinate, which enters the tricarboxylic acid cycle and participates in cellular energy metabolism (45). It plays a role in a variety of biological processes and is particularly associated with neurological disorders in the brain. Although ALDH5A1 is primarily associated with neurological function, it cannot be completely ruled out that it may indirectly affect inflammatory diseases such as AS. For example, alterations in metabolic pathways sometimes affect immune system responses and inflammatory processes (46). However, more scientific studies are needed to clarify about the causal link between ALDH5A1 and AS. Sodium valproate has been found to inhibit ALDH5A1 and is also a non-competitive direct inhibitor of brain microsomal long-chain fatty acyl coenzyme A synthetase. This inhibition reduces available amygdalenyl coenzyme A, which in turn reduces inflammatory prostaglandin production (47–49). Thus, one of the possible mechanisms for valproate treatment of AS is similar to that of NSAIDs commonly used in migraine treatment, as they are also capable of inhibiting prostaglandin production. Furthermore, the results of the PheWAS study showed that inhibition of ALDH5A1 function did not cause potential adverse effects. Therefore, ALDH5A1 is expected to be a relatively perfect target for the treatment of AS.

In contrast, plasma proteins that are negatively associated with AS in this study are AIF1 and ACOT13.AIF1 is a 17 kDa cytoplasmic protein with the ability to bind calcium and actin. AIF1 is known to induce the production of pro-inflammatory molecules, including the inflammatory factors IL-6 and TNF-α, and cause elevation of reactive oxygen species (ROS) (50). Furthermore, it has been shown that the role of AIF1 in macrophages is critical for maintaining their survival and promoting inflammatory activity (51, 52). Based on these properties, it is reasonable to assume that there may be a positive correlation between AIF1 and AS, which is an immune-mediated disease characterized by chronic inflammation. However, our results obtained through a MR study unexpectedly show a negative association between AIF1 and AS. This finding challenges our conventional understanding of the role of AIF1 in AS and suggests that the role of AIF1 in the complex network of inflammation and immune regulation may be more complex than expected. Thus, this result highlights the importance of further in-depth studies on the mechanism of AIF1’s role in AS to reveal its true impact in disease onset and progression. According to the literature currently available, no activators of AIF1 have been identified. Moreover, in the PheWAS analysis, activation of AIF1 showed potential benefits for the treatment of 13 diseases, including AS, but was accompanied by 23 adverse effects. Therefore, we need to carefully weigh the benefits and risks. ACOT13 is a key enzyme class in the human body belonging to the aldehyde dehydrogenase family. This enzyme plays a role in fatty acid oxidation by hydrolyzing fatty acyl-coenzyme A (Acyl-CoA) to produce free fatty acids (FFA) and coenzyme A (CoA) (53). Studies have shown that regulatory T cells (Tregs) tend to use fatty acid oxidation (FAO) as their main energy source and that fatty acids and their oxidation processes not only provide energy but also promote the differentiation of Tregs (54). Tregs, in turn, play a crucial role in maintaining the balance of the immune system, which is important for the prevention of autoimmune diseases by suppressing the immune response and ensuring that the response to foreign antigens and self-antigens is kept in moderation (55). In view of this ACOT13 is expected to be a new therapeutic target for AS. Base on the literature currently available, no activators of ACOT13 also have been identified. Furthermore, in the PheWAS analysis, activation of ACOT13 was shown to be potentially beneficial for the treatment of five diseases, including AS, and was not associated with adverse effects. So ACOT13 is also a relatively perfect therapeutic target.

This proteome-wide MR analysis combining pQTL and AS GWAS data reinforces the causal relationship between plasma proteins and risk of AS, providing new insights into the treatment of AS. The significant strengths of the study are the use of MR design to reduce potential confounders and bias from reverse causality, followed by the inclusion of cis-pQTLs to improve the level of evidence (cis-pQTL>trans-pQTL>eQTL), and then gene colocalization analyses to improve statistical efficacy, thus enhancing the credibility of the findings. Finally full phenotypic association analysis allowed us to explore the side effects of potential drug targets in greater depth. We are also keen to encourage other researchers to adopt the PheWAS approach to dig deeper into the potential side effects of drug targets to enrich the knowledge base of the field as a whole. However, there are some limitations in this study: 1. all GWAS participants were of European origin, which may affect the generalizability of its results; 2. although the UKP-PPP data contained 2,940 plasma proteins, only 1,908 plasma proteins were ultimately included in the MR study due to the limitation of the instrumental variables; 3. due to the limited power of the colocalization analyses, we focused our study on the cases where the joint posterior association probability (PPH3+PPH4) was equal to or greater than 0.8; 4. It may not be possible to supplement cell and animal experiments due to realistic conditions, and we will consider including these experiments in our research program or in collaboration with other laboratories to advance research in this area; 5. This study screened for causal associations between plasma proteins and AS using a MR approach but it did not utilize a genetics-led drug target prioritization method (Priority index, PI) to prioritize under-explored targets (56–58).

## 5 Conclusion

Our investigation examined the causal relationship between six plasma proteins, including AIF1, TNF, FKBPL, AGER, ALDH5A and ACOT13, and AS, providing new targets for the treatment of AS. We look forward to exploring these drug targets and their potential therapeutic approaches, as well as their impact on the treatment of ankylosing spondylitis, in more depth in future studies.

## Data availability statement

The original contributions presented in the study are included in the article/**Supplementary Material**. Further inquiries can be directed to the corresponding authors.

## Ethics statement

Ethical approval was not required for the study involving humans in accordance with the local legislation and institutional requirements. Written informed consent to participate in this study was not required from the participants or the participants’ legal guardians/next of kin in accordance with the national legislation and the institutional requirements.

## Author contributions

WZ: Formal analysis, Investigation, Methodology, Software, Visualization, Writing – original draft. PF: Formal analysis, Investigation, Methodology, Software, Visualization, Writing – original draft. CL: Formal analysis, Investigation, Methodology, Software, Visualization, Writing – original draft. XX: Data curation, Visualization, Writing – review & editing. YW: Formal analysis, Investigation, Software, Supervision, Writing – review & editing. HL: Methodology, Project administration, Resources, Writing – review & editing. HJ: Data curation, Formal analysis, Methodology, Project administration, Resources, Supervision, Validation, Visualization, Writing – review & editing. XL: Conceptualization, Data curation, Formal analysis, Funding acquisition, Investigation, Methodology, Software, Supervision, Visualization, Writing – review & editing. JL: Conceptualization, Data curation, Formal analysis, Funding acquisition, Investigation, Methodology, Software, Supervision, Visualization, Writing – review & editing.
